# Variation in chemokines plasma concentrations in primary care depressed patients associated with Internet-based cognitive-behavioral therapy

**DOI:** 10.1038/s41598-020-57967-y

**Published:** 2020-01-23

**Authors:** Pablo Romero-Sanchiz, Raquel Nogueira-Arjona, Pedro Araos, Antonia Serrano, Vicente Barrios, Jesús Argente, Nuria Garcia-Marchena, Antonio Lopez-Tellez, Silvia Rodriguez-Moreno, Fermin Mayoral, Francisco J. Pavón, Fernando Rodríguez de Fonseca

**Affiliations:** 1 Unidad de Gestión Clínica de Salud Mental. Instituto de Investigación Biomédica de Málaga (IBIMA), Hospital Regional Universitario de Málaga/Universidad de Málaga, Málaga, 29010 Spain; 2 Department of Personality, Assessment, and Psychological Treatment, School of Psychology, University of Malaga, Blv. Louis Pasteur s/n, Malaga, CP 29010 Spain; 3 Department of Psychology and Neuroscience, Dalhousie University, Life Sciences Centre, 1355 Oxford Street, PO BOX 15000, Halifax, Nova Scotia B3H 4R2 Canada; 4 Department of Endocrinology, Research Institute “La Princesa”, Hospital Infantil Universitario Niño Jesús, Madrid, Spain; 5 Department of Pediatrics, Universidad Autónoma de Madrid, Madrid, Spain; 6 CIBER Fisiopatología Obesidad y Nutrición (CIBERobn), Instituto de Salud Carlos III, Madrid, Spain; 7 CEI UAM + CSIC, IMDEA Food Institute, Madrid, Spain; 8 Unidad de Gestión Clínica Puerta Blanca, Av. Gregorio de Diego, 46, Málaga, CP 29004 Spain; 9 Unidad de Gestión Clínica Teatinos/Colonia Santa Inés, Calle Andrés Bernáldez, 12, Málaga, CP 29010 Spain; 10 Departamento de Psicobiología y Metodología de las Ciencias del Comportamiento,Facultad de Psicología, Universidad de Málaga (UMA), Málaga, Spain; 11 Institut Germans Trias i Pujol -IGTP-Campus Can Ruti, Carretera de Canyet s/n, Badalona, 08916 Spain

**Keywords:** Biomarkers, Biomarkers

## Abstract

How the presence of inflammation has repercussions for brain function is a topic of active research into depression. Signals released from immune system-related cells, including chemokines, might be indicative of active depression and can, hypothetically, serve as biomarkers of response to interventions, both pharmacological and psychological. The objective of this study is to analyze the peripheral plasma concentrations of CXCL12, CCL11, CX3CL1 and CCL2 in a cohort of depressed primary-care patients, as well as their evolution after an internet-based cognitive-behavioral intervention. The concentrations of those chemokines were measured in 66 primary-care patients with mild and moderate depression, before and after the intervention, as well as 60 controls, using multiplex immunoassays. Concentrations of CXCL12 and CCL2 were significantly higher in the clinical sample in comparison with controls. A stable multivariate discriminative model between both groups was found. Concentrations of all chemokines decreased after the internet-based psychological intervention. These findings support the implication of chemokines in depression, even in a sample of patients with mild and moderate severity. Furthermore, they demonstrate the need for further multidisciplinary research that confirms how biomarkers such as plasma chemokines can serve as a marker for depression and are sensitive to non-pharmacological interventions.

## Introduction

Depression is one of the most prevalent mental health problems throughout the world. In the last decade, it has been identified by the World Health Organization as one of the leading causes of disability worldwide^1,2^. Despite extensive empirical support for the effectiveness of both psychological and pharmacological interventions^3,4^, many patients remain resistant to treatment^5,6^. In response, current advances in neuroscience have identified additional mechanisms of the disorder’s underlying pathophysiology, leading to alternative or additional pathogenic hypotheses and therapeutic interventions^5,7^.

One of these new actors in the neurobiology of depression has emerged from studies on neuroinflammation, with the relationship between immunological-inflammatory responses and depressive symptoms becoming one of the most studied areas in current research into depression^8,9^. In this hypothesis, the activation of certain inflammatory pathways and/or immunomodulatory signaling networks is associated with the pathophysiology of depression, at least in a subset of patients^8^. Among all the proposed underlying mechanisms that associate depression with inflammation, the dysregulation of cytokines is one of the more extensively studied^10–12^. For instance, cytokines have been suggested as predictors of the antidepressant effect of exercise^13^, and meta-analysis has shown that certain types of antidepressants reduce pro-inflammatory factors such as C-reactive protein, tumor necrosis factor-α and interleukin −1β, showing some interaction between antidepressant medication, depression and inflammation^14^. Another review showed that the effects of antidepressant drugs have been consistently linked to decreased inflammation^15^. However, despite the extensive research into cytokines in this area, a related family of immune system-derived signaling proteins, the chemokines, has been relatively neglected^7^. Chemokines have been traditionally involved in the chemotactic function, attracting and modulating the function of mononuclear phagocytic cells to inflammatory focus, including the central nervous system (CNS), where they can attract monocytes to cortical areas related to psychiatric disorders, including depression and bipolar disorders^16–18^. Current research has revealed a wider function of chemokines beyond the classical chemoattractant role. Chemokines in the brain may modulate microglial cells, the CNS-resident macrophages cells. These phagocytic cells colonize the brain early in development, playing an important modulatory role in synaptic plasticity processes^19^. Chemokines are important modulators of microglial function, resulting in modulation of important plasticity events that include synaptic pruning and remodeling. In addition, chemokines participate in neurotransmission, neurogenesis and neurodevelopment. These actions might have a clear influence on the pathogenesis and clinical evolution of neurological and psychiatric disorders.

According to some studies, chemokines are implicated in the pathophysiology of depression through neurotransmitter-like and neuromodulatory effects, or the regulation of axon sprouting and neurogenesis^7,20^. As an example, the chemokine CXCL12 has been demonstrated to modulate neuronal control of serotonin dorsal raphe neurons involved in depression^21^. Recent research into the involvement of some chemokines in depression has reported alterations in circulating chemokines in clinical human samples^22^. A number of cross-sectional studies have found links between depression and cytokines such as CCL2, IL8 and CCL11^23^. However, a recent review^20^ points out that “chemokines with great mechanistic relevance including CXCL12 and CX3CL1 have been rarely reported in the existing human literature and should be included in future clinical studies.” Also, the results are relatively mixed and prospective studies are scarce^20^. Studies with a prospective methodology, in comparison with cross-sectional designs, provide a stricter control of potentially confounding variables, given that each subject acts as its own control.

## Aims of The Study

In previous studies, our research group has found that some of these chemokines – specifically, CCL2, CCL11, CX3CL1, and CXCL12 – are related with depressive symptoms in patients with cocaine^24–26^ and alcohol^27^ use disorders. Following those findings, this study has two main aims: Firstly, the relationship between depression and circulating plasma concentrations of CCL2, CCL11, CX3CL1, and CXCL12 will be tested. For that purpose, the differences in the plasma concentration of those chemokines between two samples – one of primary-care patients diagnosed with depression, and another of healthy non-depressed controls – will be tested. The differential influence of sex and antidepressant medication will also be tested. Secondly, the variations of the concentrations in plasma of these molecules in those patients before and after an internet-based cognitive-behavioral therapy (iCBT) intervention will be tested.

There are two reasons for studying the concentrations of chemokines in this particular type of patient: Firstly, in general, primary-care patients often suffer less severe depression and have fewer comorbidities than those who attend specialized units. Also, it is easier to find relatively naïve patients in terms of antidepressant prescription^28^ – although, in the present study, we will recruit both antidepressant-naïve and ISSR-treated patients. These features are particularly interesting for directly relating the concentrations of chemokines to depressive symptoms, without the interference of potentially confounding variables. Secondly, and related to the previous argument, the fact that the patients follow a psychological intervention would improve the insight into the mechanisms of influence of chemokines in depression, independently of antidepressant medication.

We hypothesized that the plasma concentrations of CCL2, CCL11, CX3CL1, and CXCL12 would be higher in the sample of depressed patients in comparison with the sample of controls. We also hypothesized that the plasma concentrations of CCL2, CCL11, CX3CL1, and CXCL12 in the sample of depressed patients would be reduced after the iCBT intervention.

## Material and Methods

### Participants and recruitment

The clinical sample (n = 66) comprised patients with a low mood-related complaint. General practitioners in primary-care settings conducted the recruitment. The patients were asked to participate in two parallel studies. The first evaluated the efficacy of a 3-month iCBT program for depressed primary-care patients, and the second studied potential biomarkers of depression. Patients that agreed to participate in both studies were included in this one. The inclusion criteria were: (a) between 18–65 years old, (b) depressive symptoms lasted at least two months, (c) major depressive disorder diagnosis, and (d) mild or moderate depression severity scores (mild: 14–19; moderate: 20–28). The exclusion criteria were the following: (a) severe mental disorder or a substance-use disorder diagnoses, (b) currently pregnant or breastfeeding, or (c) chronic infectious or inflammatory diseases were present. The patients were evaluated at baseline and post-treatment using a battery of questionnaires, and they were diagnosed using a structured interview (see below) by a trained clinical psychologist.

Patients with prescribed antidepressant medication were allowed to take part in the studies, but this medication had to have been prescribed at least four weeks before the beginning of the studies and had to remain stable during that period. If the antidepressant medication was changed or the dosage increased, patients were excluded from both studies. Nineteen patients were on prescribed antidepressants (five citalopram, five sertraline, four fluoxetine, two trazodone, two paroxetine, and one duloxetine).

The control group was formed by 60 volunteers recruited by the researchers from the hospital staff. The participants were interviewed using a screening tool to rule out the presence of psychopathological symptoms, addictions, as well as any type of medication in the last month. They were informed about the characteristics of the study and were asked to take part on a voluntary basis if they fulfilled the criteria. See Table 1 for sociodemographic and clinical data at baseline.

**Table 1:** Sociodemographic and clinical data at baseline.

*Variable*		p-*value*	*Control group* n = 60	*Patients group* n = 66
Age (yrs) [mean (SD)]		0.001^a^	36.28 (9.910)	42.56 (9.897)
Sex [n (%)]	Men	0.024^b^	31 (51.7)	21 (31.8)
	Women		29 (48.3)	45 (68.2)
Marital status [n (%)]	Single	0.000^b^	29 (50.0)	9 (13.6)
	Married/Couple		26 (44.8)	47 (70.0)
	Divorced/Separated		0 (0)	7 (10.6)
	Widow		3 (5.2)	3 (4.5)
Education [n (%)]	Primary	0.218^b^	10 (17.2)	9 (13.4)
	Secondary		20 (34.5)	34 (50.7)
	University		28 (48.3)	24 (35.8)
Employment [n (%)]	Employed	0.023^b^	47 (81.0)	41 (62.1)
	Unemployed		11 (19.0)	20 (30.3)
	Student		0 (0)	5 (7.6)
BMI(kg/m^2^) [mean (SD)]		0.892^a^	25.334 (4.655)	25.221 (4.682)
BDI (rank: 0–63) [mean (SD)]		—	—	24.687 (7.167)
Pharmacological Treatment [n (%)]	Antiinflammatories	—	—	2 (3.0)
	Antihypertensives	—	—	3 (4.5)
	Antidepressants	—	—	19 (28.8)
	Anxiolytics	—	—	26 (39.4)
	Other	—	—	—

BMI: Body mass index; BDI: Beck Depression Inventory; ^a^p-value from Student’s t-test or Mann-Whitney’s U test; ^b^p value from Fisher’s exact test or chi-square test.

### Ethics statement

All patients and participants of the control group signed written informed consent. The current study and its recruitment protocols were approved by the Regional Research and Ethics Committee of the Hospital Regional University of Málaga. Therefore, this study was conducted in accordance with the “Ethical Principles for Medical Research Involving Human Subjects” adopted in the Declaration of Helsinki by the World Medical Association.

### Intervention

**Figure 1:**
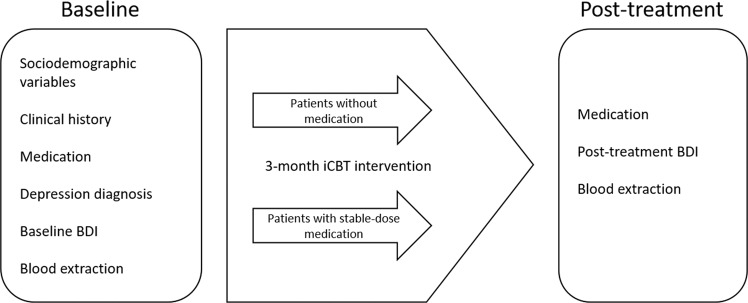
iCBT intervention Timeline.

The psychological intervention used in this study was the internet-delivered self-help program “Smiling is fun.” This program has been developed^29^ for the treatment of depressed primary-care patients among the Spanish population, and its efficacy^30^, cost-effectiveness and cost-utility^31^ have been established. “Smiling is fun” consists of 10 web-delivered sequential modules with different CBT-based techniques for coping with mild and moderate depression. The modules are as follows: (1) medication management, (2) sleep hygiene, (3) motivation for change, (4) understanding emotional problems, (5) learning to move on, (6) learning to be flexible, (7) learning to enjoy, (8) learning to live, (9) living and learning, and (10) from now on, what else? The duration of the intervention was 3 months, and the participants were assessed at baseline and post-treatment (see Fig. 1). The content of the program can be found elsewhere^32^.

### Measures

#### Beck Depression Inventory-II (BDI-II)

This questionnaire is formed by 21 items that assess the severity of depression symptoms in a multiple-choice format^33^. Different studies have shown that the BDI-II has excellent internal consistency, validity, and test–retest reliability^34,35^.

#### Structured Clinical Interview for DSM-IV Axis I Disorders–Clinician Version (SCID-CV)

The SCID is a semi-structured interview that assesses Axis I disorders from the fourth version of the Diagnostic and Statistical Manual of Mental Disorders (DSM-IV)^36,37^. This interview is the most frequently used instrument for the diagnosis of DSM disorders. In this study, the SCID-IV was used to standardize the diagnosis of major depressive disorder.

### Blood extractions and processing of plasma samples

Venous blood samples were extracted twice, one at baseline and the second one right after post-treatment interview, in 12-h-fasted conditions from 8.30–11.00 a.m. We used 10 ml K2 EDTA tubes (BD, Franklin Lakes, NJ, USA). To obtain plasma, samples were centrifuged at 2,200 g for 15 minutes (4 °C). Three tests to detect infectious diseases were conducted in each sample (exclusion criteria): HIV (Retroscreen HIV, QualPro Diagnostics-Tulip Group Ltd, Goa, India), hepatitis B (HBsAg Test, Toyo Diagnostics-Turklab Inc., Izmir, Turkey) and hepatitis C (Flaviscreen HCV, QualPro Diagnostics-Tulip Group Ltd). Each plasma sample was itemized and labeled, and samples that displayed infection following our lab safety protocols were discarded. The samples were preserved at −80 °C until the chemokines were quantified.

### Multiplex Immunoassays

Chemokine concentrations were measured using a Bio-Plex Suspension Array System 200 (Bio-Rad Laboratories, Hercules, CA, USA) with Procarta Immunoassay Kits using polystyrene beads and a Plasma Standard Diluent Kit (Affymetrix-Panomics, Santa Clara, CA, USA). This type of analysis is based on Luminex technology. At the same time, a human chemokine 6-plex panel was used to detect CXCL12, CCL11, CX3CL1, and CCL2. Characterizations were conducted following the manufacturer’s instructions. Raw data was analyzed with the software Bio-Plex Manager 4.1 (Bio-Rad Laboratories, Hercules, CA, USA). Data are expressed as picograms of protein per milliliter of plasma.

### Data analytics plan

The Shapiro–Wilk test was used to determine the normality of the scores in all the variables. The main variables that were not distributed normally were converted using a base-10 logarithmic transformation. For data expressed by means and standard deviations (SD), either Student’s t-tests or Mann–Whitney U tests were used to test differences between groups, depending on the normality of the scores. For data expressed in percentages, chi-square tests were used. Despite multiple comparisons being performed (differences in four chemokines), we decided not to use Bonferroni correction, since the study was exploratory. Receiver operating characteristic (ROC) analyses calculating the area under the curve (AUC) were used to identify predictors for differentiating between groups and to evaluate the predictive power of the logistic models. Binary logistic regression models were created that included the selected chemokines (predictors), and the goodness of fit of the models was tested with the Hosmer–Lemeshow test. A backward stepwise approach was used to restrict the model to the most predictive predictors. The pre-post analyses were carried out twice, one with the whole sample and an additional one with those patients who improved (sensitivity analysis). The criterion used for improvement was that the patient stepped down from moderate to mild or no depression, or from mild to no depression. P-values lower than 0.05 were considered statistically significant. Statistical analyses were conducted using IBM SPSS Statistics 22.0 (IBM, Armonk, NY, USA) and GraphPad Prism 6.01 (GraphPad Software, San Diego, CA, USA).

## Results

### Comparison between depressed patients and controls

Chemokine concentrations were compared using t-tests after a base-10 logarithmic transformation (Log_10_), given that their distributions were not normal. Statistically significant differences in CXCL12 and CCL2 concentrations were found between depressed patients and controls (Table 2). Given that there were age and sex differences between samples, one ANCOVA for each molecule was carried out using those plus body mass index (BMI) as covariates. Differences in CXCL12 and CCL2 remained, and the concentration of CX3CL1 was significantly higher in controls.

**Table 2:** Differences in chemokine levels between depressed patients and controls (t-test).

Concentration (ng/ml)	Patients EMM (CI) (N = 66)	Controls EMM (CI) (N = 60)	t (df)	P	ES
CXCL12	319.889 (304.789–335.738)	281.190 (267.301–295.801)	−3.688 (124)	0.000**	0.661
CCL11	125.893 (112.720–140.930)	124.451 (110.662–139.637)	−0.154 (124)	0.878	0.028
CX3CL1	1.629 (1.377–1.928)	1.892 (1.589–2.254)	1.218 (124)	0.225	0.212
CCL2	28.642 (26.853–30.479)	25.704 (24.099–27.479)	−2.306 (124)	0.023*	0.411
CXCL12	322.107 (306.196–338.844)	279.254 (264.850–294.442)	13.926 (1)	0.000*	0.103
CCL11	126.183 (112.720–141.579)	123.879 (109.901–139.959)	1.418 (1)	0.236	0.000
CX3CL1	1.492 (1.264–1.762)	2.084 (1.749–2.477)	8.194 (1)	0.005**	0.055
CCL2	28.510 (26.730–30.408)	25.823 (24.154–27.669)	4.065 (1)	0.046*	0.032

Differences in chemokine levels between depressed patients and controls controlling for age, gender and BMI (ANCOVA). EMM, Estimated marginal means; CI, Confidence intervals; T, Student’s t; ES, effect size (Cohen’s d); *p < 0.05, two-tailed; **p < 0.01, two-tailed.

In order to study the influence of sex, independent t-test analyses were carried out, splitting both samples by sex. CXCL12 was higher in depressed men and women in comparison with controls, and CCL2 was higher only in depressed men in comparison with controls (Tables S1 and S2).

Finally, a comparison between patients medicated with antidepressants, non-medicated patients and controls was carried out using an ANOVA test, showing differences in the concentrations of CXCL12 (Table 3) between non-medicated patients and controls.

**Table 3:** Differences in chemokine levels between medicated depressed patients, non-medicated depressed patients and controls (antidepressants).

Concentration (ng/ml)	Medicated EMM (CI) (N = 19)	Non-medicated EMM (CI) (N = 47)	Controls EMM (CI) (N = 60)	F (df)	P	ES
CXCL12	315.500 (280.414–355.059)	322.033 (302.901–342.295)	281.061 (268.720–293.968)	6.826 (2)	0.002*	0.100#
CCL11	123.084 (96.828–156.495)	127.116 (109.345–144.774)	124.366 (112.254–137.816)	0.045 (2)	0.956	0.001
CX3CL1	1.722 (1.352–2.193)	1.593 (1.274–1.991)	1.892 (1.586–2.257)	0.823 (2)	0.442	0.013
CCL2	29.087 (25.668–32.953)	28.431 (26.412–30.598)	25.734 (24.021–27.561)	2.693 (2)	0.072	0.042

EMM, Estimated marginal means; CI, Confidence intervals; F, Anova’s F; ES, effect size (Partial Eta squared).

^#^Difference between Non-medicated patients and Controls; *p < 0.05, two-tailed; **p < 0.01, two-tailed.

### Multivariate discriminative model between depressive and control groups

**Figure 2:**
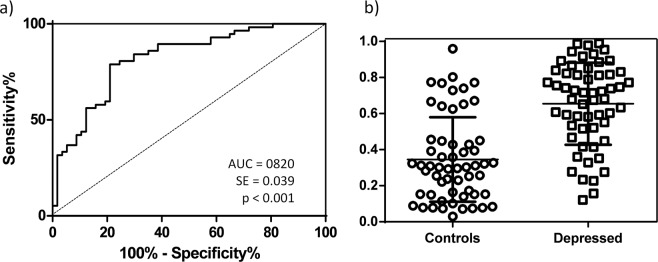
Binomial logistic regression. (**a**) ROC curve and (**b**) scatter plot of the predictive probabilities between depressed patients and controls, controlling for age, gender and BMI. AUC: Area Under the Curve; SE: Standard Error.

A model for the discrimination between depressed patients and controls was tested using binomial logistic regression analysis. The concentrations of all chemokines were used as predictors. Age, sex, and BMI were included in the first step. After five iterations, the model showed good calibration with a Hosmer–Lemeshow test (χ^2^ = 9.212; *p* = 0.325). A ROC curve was drawn, and it showed a statistically significant AUC (AUC = 0.820*; p* < 0.001; Fig. 2a), with a cut-off score of 0.462 (sensitivity = 78.95%; specificity = 78.95%). The means of the predictive probabilities of the model between depressed patients and controls (Fig. 2b) were, respectively, 0.655 (SD = 0.030) and 0.346 (SD = 0.031). The differences between groups were statistically significant (*t* = 7.138; *p* < 0.001).

### Variation in depression scores and concentration of chemokines after a psychological intervention

The mean depression score of the 41 depressed patients who completed blood extractions at baseline and post-treatment decreased significantly (*t* = 6.544; *p* = 0.000) from 23.829 (SD = 6.070) at baseline to 14.829 (SD = 8.752) at post-treatment. Almost three out of four (73.2%) participants improved after the iCBT treatment, 19.5% remained in the same category and 7.3% got worse. A total of 48.8% of the patients stepped down to no-depression scores at baseline.

The variation in chemokine concentrations was tested using a repeated-measures t-test analysis for each molecule. Statistically significant reductions between baseline and post-intervention measures were found in all molecules (Table 4). Sensitivity analyses with patients who improved (n = 30) showed the same results (Table 4). Statistically significant reductions in all chemokines were found in patients with and without antidepressant medication. Finally, splitting the sample by sex, the same reductions were found in women. In men, it was only in CX3CL1 that the decrease was not statistically significant (p = 0.057).

**Table 4:** Variation in chemokine levels before and after internet-based CBT intervention for patients with mild and moderate depression (N = 41) and those who improved (N = 30).

Concentration (ng/ml)	Pretreatment EMM (CI)	Post-treatment EMM (CI)	t (df)	p	ES
**Patients with baseline and post-treatment blood sample (N = 41)**					
CXCL12	315.500 (293.089–340.408)	158.489 (140.929–178.238)	16.120 (40)	0.000*	1.556
CCL11	125.314 (109.144–143.880)	82.414 (76.033–89.125)	6.262 (40)	0.000*	1.135
CX3CL1	1.874 (1.524–2.301)	0.508 (0.413–0.625)	9.344 (40)	0.000*	1.994
CCL2	28.907 (26.669–31.261)	18.281 (16.520–20.184)	10.461 (40)	0.000*	1.569
**Patients with baseline and post-treatment blood sample that improved (N = 30)**					
CXCL12	305.492 (282.488–329.610)	145.211 (129.122–163.305)	18.175 (29)	0.000*	2.566
CCL11	124.451 (106.170–145.881)	87.096 (79.983–95.060)	4.790 (29)	0.000*	0.994
CX3CL1	1.738 (1.342–2.249)	0.483 (0.383–0.608)	7.402 (29)	0.000*	1.957
CCL2	28.708 (26.424–31.260)	18.323 (16.218–20.749)	8.228 (29)	0.000*	1.545

EMM, Estimated marginal means; CI, Confidence intervals; T, Student’s t; ES, effect size (Cohen’s d); *p < 0.05, two-tailed; **p < 0.01, two-tailed.

## Discussion

The aim of this study was to test the relationship between depression and plasma concentrations of CCL2, CCL11, CX3CL1 and CXCL12 in mildly and moderately depressed primary-care patients, as well as the potential influence of an effective iCBT intervention on those molecules. To our knowledge, this is the first study that checks the variation in chemokine concentrations after an effective psychological intervention. Two remarkable aspects can be highlighted from the present study with regard to the biological significance of chemokines in depression. First is the demonstration that CBT-based interventions can normalize or even reduce the elevation of chemokines found in depressive patients. Because systemic inflammation can affect ascending monoamine transmitters involved in emotions and emotional learning (i.e., reward associated), CBT intervention, by reducing inflammation (including chemokines), has a biological way to improve depression-associated symptomatology^38^. In this regard, both inflammation and chemokines have been demonstrated to affect the activity and release of dopamine and serotonin in humans and experimental animals^21^. The second remarkable aspect is that the identification of the biological significance for each of the different chemokines identified might offer the possibility of helping to understand specific aspects of the biology of depression and co-morbid disorders. Further investigation is clearly needed to achieve this goal, although initial results clearly support this research line. For instance, CCL11 has been recently identified as a chemokine linking cocaine use disorders with major depression^39^.

The concentrations of CCL2 and CXCL12 were significantly higher in depressed patients, compared to controls. Neither CCL11 nor CX3CL1 concentrations were altered at baseline. These findings on CCL2 supported two recent meta-analyses describing the association of depression with alterations in plasma cytokine/chemokine profiling^10,23^. Despite the heterogeneity of the results included in those meta-analyses, the elevated concentration of CCL2 in depressed patients seems to be quite established. The results for CXCL12 also replicate a previous study that evaluates the plasma concentrations of CXCL12 of depressed patients and controls^40^. This chemokine has a tight relationship with serotonin transmission. As well as CXCL12 regulating serotonergic activity of dorsal raphe nuclei serotonergic neurons^21^, its action on peripheral T-lymphocytes is modulated by serotonin^41^. With respect to CX3CL1, contrary to what was hypothesized, its concentration was significantly higher in controls after controlling age, sex and BMI. However, this result is not entirely surprising, given that studies linking this molecule with depression are scarce^20,42^. In fact, CX3CL1 differs from other chemokines in a number of ways. For instance, in contrast to other chemokines, CX3CL1 has a membrane-bound form^7^, which is essential in neuron-glia physical interactions. In addition, CX3CL1 is present in neurons and is involved in multiple actions in the CNS, primarily in the microglial regulation state, adjusting synaptic transmission^7^. The results of splitting the clinical sample into medicated and non-medicated patients showed differences in CXCL12 between non-medicated patients and controls, and a trend between medicated patients and controls in CCL2. In both cases, the splitting of the clinical samples produced a loss in statistical power, which might have led to non-significant results. Further studies with larger samples could clarify whether there are differences in chemokine concentrations between medicated and non-medicated patients.

Interestingly, despite not all chemokine concentrations being statistically different between depressed patients and controls, the logistic regression analyses using all chemokines resulted in a model with good discrimination capacity. We are still far from the use of any molecule as a reliable biomarker, but these results support the suggestion of a distinctive pattern of chemokine concentrations between depressed patients and controls. It is important to highlight that the differences found in our study are not between controls and patients with severe depression, but between patients with mild and moderate depression, and most of them without antidepressant medication and without chronic infectious or inflammatory diseases. This is a crucial issue, in our opinion, given that previous studies included mostly patients suffering severe depression and taking antidepressants^8,10^. The fact that we found differences between controls and mostly non-medicated patients with less severe depression supports the potential mechanistic role of chemokines in depression.

As expected, depression scores decreased significantly after the iCBT intervention, similar to the findings in the randomized clinical trial conducted with this intervention^30^. In addition, chemokine concentrations significantly decreased after the iCBT intervention, even showing large effect sizes. These results were also significant and showed large effect sizes with the patients who improved after the intervention, in patients with or without antidepressant medications and in women. In men, CX3CL1 showed a trend, which could be attributed to a decrease in statistical power due to the reduction of the sample size (n = 11). These results support the suggestion that changes in depressive symptoms are associated with changes in chemokine concentrations, but also that those changes are not necessarily related to a pharmacological intervention. This is not the first study that finds neuroinflammatory changes after a psychological intervention, for instance, in cytokines^43,44^. These results also align with research suggesting that certain biomarkers might help to stratify patients that supposedly fall into the same diagnostic categories^45^. Certain initiatives, such as research domain criteria^46^, support the idea that biological markers might be used to identify treatments that could be particularly helpful for patients with specific characteristics. This study showed initial evidence supporting the existence of different chemokine concentrations in depressed patients depending on variables such as sex, or if they are taking antidepressant medication. It is well known that antidepressant medication changes inflammatory markers^15^, as does exercise^47^, psychotherapy^43^ and CBT, in comparison with other psychological interventions^44^. Further research should address if these changes are found in all empirically based psychotherapies, in patients with different degrees of severity or in other disorders. This should encourage multidisciplinary research to improve our knowledge of depression and the treatments that we can offer to those patients.

These results are in line with those found when comparing depressed patients and controls for CXCL12 and CCL2, but not for CCL11 and CX3CL1. The concentration of CCL11 was non-significantly higher in depressed patients in comparison with controls. However, CX3CL1 was non-significantly lower in depressed patients in comparison with controls, and reached statistical significance after controlling for age, sex and BMI. This means that the discrepancy in CCL11 could merely be a matter of statistical power, but the results for CX3CL1 seem to be in the opposite direction. This difference could be due to the particularities of CX3CL1 listed above and leads to very relevant questions that need further exploration with larger samples and, maybe, a different methodology (see below). Nonetheless, these discrepancies might be attributed to differences between the samples. As mentioned above, more prospective studies are needed^20^, because they provide stricter control of potentially confounding variables, and consequently provide sounder results.

The main limitation of this study is the absence of a second measure of chemokine concentrations in the control group. However, given that the recruitment criteria assured that the referred patients had consulted for a mood complaint, that antidepressant medication was stable during the study, and that patients with somatic illnesses were excluded, we believe that the results are strong and offer compelling evidence supporting the association between chemokines and depression. Another limitation relates to the chemokines selected, which constitute a small selection of the large class of chemokine signals. Further studies are needed to demonstrate that other chemokines, such as CCL4, CXCl4, CXCL7 and CXCL8, proposed as being altered in depression are also sensitive to CBT interventions. Another limitation is that the clinical sample is too small for stratification, so potentially important secondary analyses must be replicated in further, larger studies. For instance, the differences in the concentration of CXCL12 between non-medicated depressed patients and controls should be replicated using larger samples. In addition, other potentially relevant analysis could be carried out with bigger samples, analyzing the influence of different antidepressants, depression severity or the predominant types of symptoms (somatic, cognitive or behavioral). Another limitation might be the features of the clinical sample – that is, primary-care patients with mild or moderate depression. The results cannot be directly extended to other populations and should be replicated in samples with other characteristics, as with any other scientific finding. In conclusion, we believe that to find biological correlates of depressive symptoms after a psychological intervention in a sample with non-severely depressed patients and controlling the influence of medication should encourage further, larger studies, which, hopefully, might confirm these findings and improve our knowledge of depression and the way it is treated.

## Supplementary information

Supplementary Information.

## References

[1] Mathers CD, Loncar D (2006). Projections of Global Mortality and Burden of Disease from 2002 to 2030. PLoS Med..

[2] World Health Organization. *The Global Burden of Disease: 2004 Update*. (2008).

[3] National Institute for Health and Care Excellence. *Depression in adults: Recognition and management. Clinical guideline [CG90]* (2016). 31990491

[4] Cuijpers P (2013). The efficacy of psychotherapy and pharmacotherapy in treating depressive and anxiety disorders: A meta-analysis of direct comparisons. World Psychiatry.

[5] Więdłocha M (2017). Effect of antidepressant treatment on peripheral inflammation markers – A meta-analysis. Prog. Neuro-Psychopharmacology Biol. Psychiatry.

[6] Cuijpers P, Gentili C (2017). Psychological treatments are as effective as pharmacotherapies in the treatment of adult depression: a summary from Randomized Clinical Trials and neuroscience evidence. Res. Psychother. Psychopathol. Process Outcome.

[7] Stuart MJ, Singhal G, Baune BT (2015). Systematic Review of the Neurobiological Relevance of Chemokines to Psychiatric Disorders. Front. Cell. Neurosci..

[8] Köhler, C. A. *et al*. Peripheral Alterations in Cytokine and Chemokine Levels After Antidepressant Drug Treatment for Major Depressive Disorder: Systematic Review and Meta-Analysis. *Mol. Neurobiol*. 1–12, 10.1007/s12035-017-0632-1 (2017). 10.1007/s12035-017-0632-1 28612257

[9] Slavich GM, Irwin MR (2014). From stress to inflammation and major depressive disorder: a social signal transduction theory of depression. Psychol. Bull..

[10] Köhler CA (2017). Peripheral cytokine and chemokine alterations in depression: a meta-analysis of 82 studies. Acta Psychiatr. Scand..

[11] Ye Gang, Yin Guang Zhong, Tang Zhen, Fu Jia Lin, Chen Jie, Chen Shan Shan, Li Jia, Fu Tian, Yu Xin, Xu Dong Wu, Yao Jeffrey K., Hui Li (2018). Association between increased serum interleukin-6 levels and sustained attention deficits in patients with major depressive disorder. Psychological Medicine.

[12] Glaus J (2018). Mood disorders and circulating levels of inflammatory markers in a longitudinal population-based study. Psychol. Med..

[13] Rethorst CD (2013). Pro-inflammatory cytokines as predictors of antidepressant effects of exercise in major depressive disorder. Mol. Psychiatry.

[14] Eyre HA, Lavretsky H, Kartika J, Qassim A, Baune BT (2016). Modulatory Effects of Antidepressant Classes on the Innate and Adaptive Immune System in Depression. Pharmacopsychiatry.

[15] Lee, C.-H. & Giuliani, F. The Role of Inflammation in Depression and Fatigue. *Front. Immunol*. **10**, (2019). 10.3389/fimmu.2019.01696 PMC6658985 31379879

[16] Garré JM, Silva HM, Lafaille JJ, Yang G (2017). CX3CR1+ monocytes modulate learning and learning-dependent dendritic spine remodeling via TNF-α. Nat. Med..

[17] Torres-Platas SG, Cruceanu C, Chen GG, Turecki G, Mechawar N (2014). Evidence for increased microglial priming and macrophage recruitment in the dorsal anterior cingulate white matter of depressed suicides. Brain. Behav. Immun..

[18] Pantazatos SP (2017). Whole-transcriptome brain expression and exon-usage profiling in major depression and suicide: evidence for altered glial, endothelial and ATPase activity. Mol. Psychiatry.

[19] Ransohoff RM, Khoury J (2016). El. Microglia in Health and Disease. Cold Spring Harb. Perspect. Biol..

[20] Stuart MJ, Baune BT (2014). Chemokines and chemokine receptors in mood disorders, schizophrenia, and cognitive impairment: A systematic review of biomarker studies. Neurosci. Biobehav. Rev..

[21] Heinisch S, Kirby LG (2010). SDF-1α/CXCL12 enhances GABA and glutamate synaptic activity at serotonin neurons in the rat dorsal raphe nucleus. Neuropharmacology.

[22] Leighton SP (2017). Chemokines in depression in health and in inflammatory illness: a systematic review and meta-analysis. Mol. Psychiatry.

[23] Eyre HA (2016). A meta-analysis of chemokines in major depression. Prog. Neuro-Psychopharmacology Biol. Psychiatry.

[24] Araos P (2015). Plasma profile of pro-inflammatory cytokines and chemokines in cocaine users under outpatient treatment: influence of cocaine symptom severity and psychiatric co-morbidity. Addict. Biol..

[25] Pedraz M (2015). Plasma Concentrations of BDNF and IGF-1 in Abstinent Cocaine Users with High Prevalence of Substance Use Disorders: Relationship to Psychiatric Comorbidity. PLoS One.

[26] Maza-Quiroga R (2017). Evaluation of plasma cytokines in patients with cocaine use disorders in abstinence identifies transforming growth factor alpha (TGFα) as a potential biomarker of consumption and dual diagnosis. PeerJ.

[27] García-Marchena, N. *et al*. Plasma Chemokines in Patients with Alcohol Use Disorders: Association of CCL11 (Eotaxin-1) with Psychiatric Comorbidity. *Front. Psychiatry* **7**, (2017). 10.3389/fpsyt.2016.00214 PMC5242327 28149283

[28] Aragonès E, Piñol JL, Labad A, Folch S, Mèlich N (2004). Detection and Management of Depressive Disorders in Primary Care in Spain. Int. J. Psychiatry Med..

[29] López-del-Hoyo Y (2013). Low intensity vs. self-guided internet-delivered psychotherapy for major depression: a multicenter, controlled, randomized study. BMC Psychiatry.

[30] Montero-Marín J (2016). An Internet-Based Intervention for Depression in Primary Care in Spain: A Randomized Controlled Trial. J. Med. Internet Res..

[31] Romero-Sanchiz P (2017). Economic evaluation of a guided and unguided internet-based CBT intervention for major depression: Results from a multi-center, three-armed randomized controlled trial conducted in primary care. PLoS One.

[32] Botella Arbona, C., Garcia-Palacios, A., Baños, R. & Quero, S. *Tratamiento psicológico de la depresión: aplicación presencial y online*. (Editorial Síntesis, 2016).

[33] Beck, A. T., Steer, R. A. & Brown, G. *Beck Depression Inventory-II*. (Psychological Corporation, 1996).

[34] Beck AT, Steer RA, Ball R, Ranieri WF (1996). Comparison of Beck Depression Inventories-IA and-II in Psychiatric Outpatients. J. Pers. Assess..

[35] Dozois DJA, Dobson KS, Ahnberg JL (1998). A psychometric evaluation of the Beck Depression Inventory–II. Psychol. Assess..

[36] First, M. B., Spitzer, R. L., Gibbon, M. & Williams, J. B. W. *Structured Clinical Interview for DSM-IV-TR Axis I Disorders, Patient Edition (SCID-I/P, 11/2002 revision). Biometrics Research* (New York State Psychiatric Institute, 2002).

[37] American Psychiatric Association. *Diagnostic and Statistical Manual of Mental Disorders, Washington, DC, 4th Edition-Text Revised* (2000).

[38] Petrulli JR (2017). Systemic inflammation enhances stimulant-induced striatal dopamine elevation. Transl. Psychiatry.

[39] García-Marchena N (2019). Inflammatory mediators and dual depression: Potential biomarkers in plasma of primary and substance-induced major depression in cocaine and alcohol use disorders. PLoS One.

[40] Ogłodek EA, Szota A, Just MJ, Moś D, Araszkiewicz A (2014). Comparison of chemokines (CCL-5 and SDF-1), chemokine receptors (CCR-5 and CXCR-4) and IL-6 levels in patients with different severities of depression. Pharmacol. Reports.

[41] Magrini E, Szabò I, Doni A, Cibella J, Viola A (2011). Serotonin-Mediated Tuning of Human Helper T Cell Responsiveness to the Chemokine CXCL12. PLoS One.

[42] Merendino RA (2004). Involvement of fractalkine and macrophage inflammatory protein-1 alpha in moderate-severe depression. Mediators Inflamm..

[43] Dahl J (2016). Recovery from major depressive disorder episode after non-pharmacological treatment is associated with normalized cytokine levels. Acta Psychiatr. Scand..

[44] Moreira FP (2015). The effect of proinflammatory cytokines in Cognitive Behavioral Therapy. J. Neuroimmunol..

[45] Strawbridge R, Young AH, Cleare AJ (2017). Biomarkers for depression: recent insights, current challenges and future prospects. Neuropsychiatr. Dis. Treat..

[46] Insel T (2010). Research Domain Criteria (RDoC): Toward a New Classification Framework for Research on Mental Disorders. Am. J. Psychiatry.

[47] Eyre HA, Papps E, Baune BT (2013). Treating depression and depression-like behavior with physical activity: An immune perspective. Front. Psychiatry.

