# Supplementary material for: Variation in chemokines plasma concentrations in primary care depressed patients associated with Internet-based cognitive-behavioral therapy
Source: Sci Rep. 2020 Jan 23;10:1078. doi: 10.1038/s41598-020-57967-y (PMC6978323; doi:10.1038/s41598-020-57967-y)
Supplement: Supplementary file 1 — Supplementary Information. [file 41598_2020_57967_MOESM1_ESM.docx]

**Title:** Variation in chemokines plasma concentrations in primary care depressed patients associated with Internet-based cognitive-behavioral therapy.

**Authors:** Pablo Romero-Sanchiz, Raquel Nogueira-Arjona, Pedro Araos, Antonia Serrano, Vicente Barrios, Jesus Argente, Nuria Garcia-Marchena, Antonio Lopez-Tellez, Silvia Rodriguez-Moreno, Fermin Mayoral, Francisco J Pavón, Fernando Rodríguez de Fonseca.

| Table S1. Differences in chemokine levels between depressed patients and controls by gender (Male). | | | | | |
| --- | --- | --- | --- | --- | --- |
| Concentration  (ng/ml) | Patients  EMM (CI) (N=21) | Controls  EMM (CI) (N=31) | t (df) | p | ES |
| CXCL12 | 328.852 (304.789- 354.813) | 281.838 (264.850- 299.916) | -3.192 (50) | .002* | 0.915 |
| CCL11 | 147.570 (127.643-135.519) | 129.718 (115.080-145.881) | -1.395 (50) | .167 | 0.396 |
| CX3CL1 | 1.153 (0.835-1.592) | 1.799 (1.380- 2.344) | 1.964 (50) | .059 | 0.577 |
| CCL2 | 31.045 (27.925- 34.514) | 24.774 (22.750-27.039) | -3.624 (50) | .001* | 0.975 |
| EMM, Estimated marginal means; CI, Confidence intervals; T, Student’s t; ES, effect size (Cohen’s d); * p < 0.05, two-tailed; ** p < 0.01, two-tailed | | | | | |

| Table S2. Differences in chemokine levels between depressed patients and controls by gender (Female). | | | | | |
| --- | --- | --- | --- | --- | --- |
| Concentration  (ng/ml) | Patients  EMM (CI) (N=45) | Controls  EMM (CI) (N=29) | t (df) | p | ES |
| CXCL12 | 316.228 (296.483-337.287) | 280.543 (258.821- 303.389) | -2.337 (50) | .022* | 0.577 |
| CCL11 | 116.950 (100.231- 136.458) | 119.124 (98.175- 144.211) | 0.140 (50) | .889 | 0.034 |
| CX3CL1 | 1.914 (1.592- 2.301) | 1.995 (1.584-2.512) | 0.286 (50) | .779 | 0.065 |
| CCL2 | 27.542 (25.468- 29.785) | 26.792 (24.266- 29.512) | -0.453 (50) | .652 | 0.109 |
| EMM, Estimated marginal means; CI, Confidence intervals; T, Student’s t; ES, effect size (Cohen’s d); * p < 0.05, two-tailed; ** p < 0.01, two-tailed | | | | | |
